# Reports of Societies

**Published:** 1874-06

**Authors:** 


### Reports of Societies.

*Transactions of the "Chicago Society of Physicians and Surgeons." Meeting, March* 23, 1874. *Reported by* PLYM. S. HAYES, M.D.

The Society met as usual in the parlor of the Grand Pacific Hotel, the President in the chair. The minutes of the preceding meeting were read and approved.

Dr. C. T. Parkes was unanimously elected to membership, and the names of Drs. R. E. Starkweather, W. H. Warn, and E. P. B. Wilder, were presented for membership.

Dr. F. H. Davis read a clinical report of operations on the mouth, describing Dr. I. Manly's mode of preventing hæmorrhage and producing anæsthesia simultaneously.

Dr. Henrotin exhibited an hypertrophied heart, and gave the following details of the case: The patient; a laborer of foreign birth, and previous good health, with the exception of the occurrence of pneumonia about one year prior to his death; of temperate habits; weighing one hundred and seventy pounds, fell down while at work and died in three minutes. He had been able to sleep in any position, had not suffered from dyspnœa and no dropsical effusion existed elsewhere than in the pericardium. He had been engaged in labor for ten hours previous to his death, but had been unemployed for months before this occasion. Upon opening the chest the pericardium was discovered to be enormously distended, and pushed forward into the opening. It contained about fifteen ounces of clear serum, and exhibited no traces of inflammatory action. The heart was found to be enormously enlarged, weighing thirty-two ounces; and its right side was distended with dark fluid blood—the right auricle bulging in the shape of a tumor, and measuring transversely, when opened, ten inches. The aortic semi-lunar valves were rigid and fixed from a cretaceous deposit. There were no cardiac nor vascular clots. The lungs were engorged.

A discussion resulted as to the direct cause of death, in which several participated.

Dr. Bartlett read next the "Annual Report of the Section on

Pathology," separate reports having been prepared by the other members of the section, on the pathology of special disorders. The report embraced all such material as possessed new or interesting features, which had appeared in the Medical periodicals of the preceding year.

The Secretary read a communication from Dr. Wm. E. Quine, of the Committee of Arrangements of the Illinois State Medical Society, requesting information regarding the views of those present on the subject of entertaining the State Association. On motion, it was resolved, that "this Society desires to extend its aid to the Committee of Arrangements, in providing a suitable entertainment for the Illinois State Medical Society, during the approaching convention in next May."

In furtherance of a motion of Dr. Jno. E. Owens, providing that every member of the Society, connected with the staff of a Hospital, be added to the "Committee on Clinical Reports," (adopted March 9, 1874.) a list of names of such members as should be added to the committee, was presented.

The Society then adjourned.

---

***Transactions of the Chicago Society of Physicians and Surgeons.***—*Meeting of April* 13, 1874.

The Society met as usual at the Grand Pacific Hotel, the President in the chair. The minutes of the preceding meeting were read and approved. On favorable report of the Censors, Drs. R. E. Starkweather, W. H. Warn and E. P. B. Wilder, were unanimously elected to membership.

Dr. W. A. Harvey was then nominated for membership by Dr. Jackson; Dr. P. Rofe, by Dr. Simons; and Dr. G. H. Chapman, by Dr. Emmons.

The report on Surgical Pathology, prepared by Dr. Montgomery, was deferred in consequence of the number present prepared to report upon the hypodermic use of ergotine in the treatment of uterine fibroids.

Dr. A. Reeves Jackson then made the following Report:

*On the Treatment of Fibrous Tumors of the Uterus by Hypodermic Injection of Ergotine; with Cases*, from the Woman's Hospital of the State of Illinois.

There can be no longer any reasonable doubt as to the efficacy of ergotine in the treatment of certain abnormal growths of the uterus. The power of the drug to increase the contractions of the organ in parturition has long been known; and its influence in checking or diminishing discharges of blood in the menorrhagia and metrorrhagia after parturition, has also been demonstrated by MM. Ruben and Zente, and others. It was a knowledge of these and similar facts, doubtless, that induced Hildebrandt to employ it for the purpose of controlling the hemorrhages caused by the presence of uterine fibroids.

Hildebrandt's paper, published originally in the *Gazette Hebdomadaire*, November 27, 1872, contained an account of eight cases of fibrous tumor of the womb, treated by the subcutaneous injection of ergotine. The success of the treatment was highly gratifying. From a *resume* published in the *Half-Yearly Abstract of the Medical Sciences*, for January, 1873, we learn that in one case a tumor which had extended beyond the umbilicus had disappeared; in another, a tumor which had reached as far as the false ribs was much diminished in size, so that it descended to below the umbilicus; and in four cases, where the treatment had not been so thorough, there was improvement in both the local and general conditions. Under the use of the remedy, menstruation became less abundant, less irregular, less prolonged, and, especially, less painful.

Having, during the past year, had several opportunities of testing the value of this plan of treatment, I am able to corroborate the conclusions arrived at by Dr. Hildebrandt, and am induced to publish the result for the benefit of those who may be desirous of giving the remedy a trial.

Thus far, I have used the ergotine in five cases for a sufficiently long time to enable me to estimate its effect. Two of these were in hospital, and the remaining three, in private practice. In addition to these, I am also using it in four other cases, but in these the time has been too short to enable me to pronounce an opinion as to the result.

CASE I. *Fibrous Tumor of the Uterus—Excessive Hemorrhage and Pain. Improvement* under the use of the Subcutaneous Injection of Ergotine, but no Diminution of the Tumor.*

Was called to see Maria W., the subject of this case, in the early part of June, 1873. She came to the city from her home in Iowa, accompanied by her husband, for the purpose of having a surgical operation performed for the removal of an abdominal tumor, which had been diagnosticated as ovarian.

Her history is as follows: She is thirty-two years old, and has been married twelve years; always had good health until three years ago, when she had a miscarriage at the fourth month, the result of her only pregnancy. Prior to that event there had been nothing unusual as regarded menstruation, which had commenced at the age of fifteen, and had always been regular and in every way normal. The first flow subsequent to the miscarriage had occurred at the end of five weeks, and was so profuse as to alarm her, and she was obliged to go to bed. To use her own words, "it fairly ran" from her. A physician who was called in checked it by using astringent vaginal tampons. At that time she was a large, robust woman, weighing 150 pounds. Her weight is now reduced to less than 100 pounds. Since the time mentioned, she has had a number of similar attacks of hemorrhage, which have greatly prostrated her. They recur so irregularly that she has lost all count of her menstrual periods. She has rarely passed two weeks without having a flow lasting from three to seven days. Within the past year she has had excessive pain accompanying the discharges, so that she has been obliged to use opium, sometimes in frequent and enormous doses. Her appetite has been fair, and her bowels regular. She has been troubled latterly with palpitation of the heart, which is increased by exercise or any quick movement. The urine is passed in large quantity, is clear and colorless, but there is no irritability of the bladder.

A tumor of irregular outline is found occupying the lower part of the abdomen, and is traceable into the pelvis. It extends more than an inch above the umbilicus, and reaches to the pelvic brim on each side. It is dense, painless, and free from fluctuation, and cannot be moved in any direction by any justifiable amount of force. A vaginal examination shows that it also fills the pelvic cavity, and forces the os uteri firmly up behind the symphysis pubis. A flexible catheter mounted on a wire and pushed into the cervix, marks a depth of six and a half inches. A metallic sound cannot be introduced more than an inch.

As the case was clearly not a proper one for surgical interference, I advised the use of ergotine by hypodermic injection.

The patient returned to her home on the 15th of June, and the injections were commenced on the 18th. They were given by her husband, to whom I had furnished a syringe and the ergotine solution, and whom I had instructed in their use.

In a letter, dated Sept. 8th, Mr. W. informed me that his wife was much improved. Her general health was better, and she was able to walk out daily. There was no diminution in the size of the tumor, so far as he could determine, although his wife thought it was smaller. The hemorrhages were very much reduced in number and amount. In another letter, dated March 2, he says: "My wife is about the same as when I wrote you last fall. The bleedings do not come on oftener than once in three or four weeks, and do not last more than three or four days. There are some clots, but they are small and stringy. We stopped using the injection about six weeks, but the bleeding was worse, and we returned to it. She only takes one or two doses of laudanum now, on the first day of the discharge."

CASE II.—*Tumor of the Posterior Uterine Wall—Hypodermic Injection of Ergotine—Disappearance of Tumor.*

Rachel G., a mulatto, twenty-nine years of age, was admitted to the Woman's Hospital of the State of Illinois, July 2, 1873.

She commenced menstruating at thirteen, and was married at twenty-one. She had a miscarriage seven years ago, and has one child six years old. Has not been pregnant since the birth of the latter. Menstruation returns regularly every twenty-four days, lasts four days, and is not attended by unusual pain. About one year ago her health began to fail, and about the same time she discovered a "lump" in the left lower side of the abdomen, which at times was tender under pressure. This enlargement has steadily increased in size, and has assumed a more central position in the body.

Her general health seems very much impaired. Her appetite is poor, bowels constipated, and she does not sleep well. She complains of much pelvic pain and bearing-down when she walks; pulse small, weak and irregular. She is anæmic, and states that the urine is habitually colorless. Since last April has been confined to bed the greater part of the time.

On examination, the abdominal walls are found to be excessively tense and unyielding. A tumor with irregular outline is felt above the symphysis pubis, extending half way to the umbilicus. It is hard, nodulated, almost immovable, and painless. Subsequent exploration by sponge-tent and sound, shows that the tumor is a uterine fibroid of the posterior wall. The os and vaginal portion of the cervix are normal. The sound penetrates four inches.

It is decided by the hospital staff to make trial of the hypodermic injection of ergotine, but inasmuch as a menstrual period is expected in a few days, it is deemed advisable to defer the treatment until after the cessation of the flow.

July 19th. The discharge ceased two days ago, and the use of the ergotine is commenced to-day, the point selected for the introduction of the syringe being the abdominal wall, directly over the centre of the tumor. The treatment was repeated daily to August 11th, when, owing to an attack of gastric disturbance, it was discontinued until the 17th, at which time it was recommenced, and continued thence daily to August 31st, when it was again suspended by the occurrence of menstruation. The discharge lasted three days, was very scanty, and was attended by much less pain than usual.

September 3rd the injections were resumed, and continued daily to October 16th, when menstruation again appeared, the flow this time lasting four days. They were not used afterwards. During the first few days of the treatment each injection was followed within an hour by pain referred to the uterus. Occasionally, too, it was followed by vomiting. On the 22nd of July, four days after the commencement of the treatment, there was an evident decrease in the size of the tumor; and on the 6th of August an examination by abdominal palpation failed to discover its existence above the pubic bone.

Another very marked effect of the drug was shown in the promptnesss with which it lengthened the inter-menstrual period. For nearly a year this had not exceeded twenty days, but the first interval after using the ergotine it was extended to forty-five days.

The flow continued but three days, and was small in amount, the patient using only three napkins, whereas she had used twelve during the period immediately preceding. The second interval was forty-three days, and the third, thirty-one.

On November 22nd, a careful examination was made by Drs. Hurlbut, Lyman and myself, and the tumor was no longer discoverable. The only traces left of it were a thickened condition of the lower portion of the body, and supra-vaginal portion of the cervix of the uterus, together with some perimetric cellular induration. The sound entered three inches.

December 20th the patient again reports at the dispensary for examination. Her general health has been thoroughly re-established, and she is able to attend to all her household duties. The condition of the pelvic organs remains the same as at the last examination.

CASE 3. *Fibrous Tumor of the Posterior Wall of the Uterus—Hypodermic Injection of Ergotine—Diminution of the Size of the Tumor.*

Elizabeth W., colored, aged 36 years; entered the Woman's Hospital August 9, 1873, and gave the following history: Menstruation began at thirteen; she married at eighteen, and has one child seventeen years old. Her health was good until about two years ago, when she first noticed a tumor in the lower part of the abdomen. Prior to this discovery, however, menstruation had become more frequent and irregular, and for many years she has had leucorrhœa. She has had three attacks of sickness which were called inflammation of the bowels, the first occurring four years ago, and the last and most severe one two years ago. The last attack compelled her to remain in bed two months. Menstruation has become very profuse, returning every third week, and lasting eight or nine days. The discharge is attended with much pain. Its last appearance was August 1st. She also has constant leucorrhœal discharge, thick and tenacious in character, and of a greenish color. Her bowels are very much constipated, and emptied with pain and difficulty.

She is a large, fleshy woman, and does not present the appearance of one in ill health. The abdomen is occupied at its lower part by a smooth, hard, roundish, painless tumor, extending from one inch above the umbilicus to the symphysis pubis, behind which it is felt passing into the pelvis. By vaginal examination the tumor is found to occupy the upper portion of the pelvic cavity, and to press strongly against the rectum. The os uteri is drawn up behind the pubic arch, but both it and the vaginal portion of the cervix are otherwise normal. The sound enters to the depth of five inches and five-eighths. The tumor is imbedded in the posterior wall.

The hypodermic injection of ergotine was commenced August 12th. The solution used in this case was composed as follows: ergotine, 2 drams; glycerine, 1 dram; distilled water, 5 drams. Of this, 12 minims were injected into the abdominal wall about one inch below the umbilicus.

It was followed by immediate pain, which continued several days, and finally resulted in the formation of an abscess. Owing to this mishap the injection was not repeated until the 19th. It was then used again, and continued daily without any unpleasant effect to September 4th, when menstruation appeared.

The discharge ceased on the 10th, and the treatment was resumed on the 11th, and continued every day to September 31st, when menstruation again appeared, and lasted six days. The injections were recommenced at the cessation of the flow, and continued to October 18th, when they were stopped on account of the inability of the patient to make a daily visit to the hospital, she having been an out-patient for some weeks.

From that time forward she took daily, by the mouth, the same dose as had been injected. The next menstrual period occurred the last week in October.

On November 22d an examination was made. The tumor was felt reaching to a level with the umbilicus, and the sound measured a depth of five inches.

Feb. 4, 1874. The last menstruation (exact date not attainable) lasted five days, and was less profuse and less painful than for many months before. The upper boundary of the tumor is now one inch below the umbilicus, and the sound enters to the depth of four and one-quarter inches. Patient is still under treatment.

April 8. Made an examination. The tumor is two and a half inches below the umbilicus; the sound enters four inches.

CASE 4.—*Fibroid of Anterior Wall of Uterus—Hypodermic Injections of Ergotine—Improvement of all the Symptoms, and Great Diminution of Tumor.*

Mary H., a dressmaker, forty-two years of age, unmarried; was first seen Sept. 20, 1873. She commenced menstruating at fourteen, and has been regularly unwell every four weeks, until within the past fifteen months, during which period it has become irregular, and more frequent—the interval rarely exceeding fourteen days. It has always been rather profuse than otherwise, but latterly this peculiarity has greatly increased, so that during the time of the flow she is frequently obliged to use four or five napkins daily. The length of the menstrual period has also increased, from four days to seven or eight. She passes many large clots with expulsive pains. There is some irritability of the bladder at times, which is increased at the menstrual period. The appetite is good, and the bowels inclined to be constipated.

An examination discloses the presence of an abdominal tumor, extending downwards into the pelvis, from a point one inch below the umbilicus. It is irregularly ovoid in shape, with a distinct nodular projection on the right side, producing a decided bulging to the right of the median line. Exploration by the vagina and rectum reveals the fact that a large tumor, involving the anterior wall of the body and cervix of the womb, nearly fills the pelvic cavity. So firmly is the os pressed back against the rectum, that I find it impossible to pass even the smallest sized metallic sound. With much difficulty, after forcing the womb upwards by hypogastric pressure, I at last succeeded in passing a small elastic bougie to the depth of four inches, but felt convinced that this was not a sufficient test of the depth of the uterine cavity.

Oct. 9. A sanguineous discharge, lasting eight days, ceased yesterday morning, and the subcutaneous injection of ergotine is commenced to-day, the point selected for operation being the abdominal wall, a little to the right of the linea alba, and over the most prominent part of the tumor. It is repeated daily in this locality for twelve days, when, in consequence of some soreness having been produced, it is used near the umbilicus.

On November 5, the patient commenced menstruating, and the injections were discontinued. The flow, which was less profuse than for many months, lasted six days, during which time she remained in bed or on a lounge.

November 13. The injections were recommenced, and continued daily. On the 20th, a careful examination showed that the tumor had greatly decreased in size, its upper boundary not being discoverable more than one inch above the pubic bone. The examination (a sound was not used) was followed by a sanguineous discharge, lasting one day. It was not attended by pain.

December 8th. Patient has just ceased menstruating. The flow commenced on the 3d, and has been natural as regards quantity and quality. She suffered a good deal of pain during the first thirty-six hours, but had none during the subsequent days of the period. The fundus of the womb, still presenting an irregular outline, with a right lateral prominence, is felt just on a level with the superior border of the pubic bone.

From this time forward, the injections were used irregularly, at intervals of two, three or four days. The patient's general health having greatly improved, she felt less interest than formerly in keeping up the treatment. The last examination was made Feb. 6th, and at that time there was no perceptible change in the condition of the womb since the preceding one. It still rose to the level of the pubic ridge. Menstruation had been regular and comparatively painless, and she regarded herself as wholly recovered.

CASE 5.—*Subperitoneal Tumor of the Uterus, Complicated with Vaginismus—Hypodermic Injection of Ergotine—No Improvement.*

Ellen P., a Canadian, aged twenty-eight years, is unmarried. She did not menstruate until nearly nineteen. She was sickly during infancy and childhood. At fifteen there seemed to be an attempt at menstruation, indicated by recurring pains about the pelvis and loins, headache and nausea. These attacks occurred at intervals of four or five weeks and during their continuance she had a rather copious leucorrhœa. Treatment at that time, continued during the greater part of a year, was employed to establish the menstrual function, but without avail, and the molimenal symptoms gradually subsiding, it was finally given up. At nineteen, these symptoms again appeared, and menstruation was at length established, although the discharge has always been scanty, pale, and accompanied by great pain. It has never lasted more than three days, and has never returned in less time than four weeks.

Nearly six years ago she noticed a slight fullness or lump above the pelvis, which she could push from side to side, and which seemed to her to be about the size of a hen's egg. It varied in sensitiveness, being at times quite tender, and at others not at all so. Once she had severe pain in the part, lasting several days, and since that time it has not been so freely movable as before. It has enlarged somewhat, but very slowly.

She is pallid and thin; appetite poor, and bowels constipated. She has constant leucorrhœal discharge, somewhat irritating in character, but never very profuse.

On examination, I find the abdominal walls very thin, and the hypogastric protuberance is clearly evident by inspection. It is inclined slightly to the left side of the median line. An attempt to pass the finger into the vagina produces such excessive pain and spasmodic contraction, that I am obliged to administer chloroform in order to complete the examination.

By vagino-abdominal touch, the uterus is found occupying its normal position in the pelvis, but less freely movable than usual. Springing from the left side of the fundus I could clearly make out the presence of a tumor, having a tolerably broad base of attachment. It moved consentaneously with the womb. The sound entered to the depth of three inches.

The patient had heard of the treatment of uterine tumors by means of the ergotine injections, and was anxious to have a trial of it made in her case. I consented to the suggestion, but, at the same time, expressed the great doubt I felt as to its efficacy in the treatment of growths situated as this was.

Fifty-two injections were used in all, the first being given August 4. They were given daily, except in two instances, when an interval of two days occurred. They were also suspended during the menstrual periods. These latter were apparently more painful than before the treatment. An examination, made October 1st, showed no diminution in the size or condition of the uterus, and the injections were no longer used.

*Remarks.* The solution used by Hildebrandt was composed of three parts of ergotine, 7.5 parts of glycerine, and 7.5 parts of water. He preferred this on the ground that it produced much less pain than the solution which had previously been used by Langenbeck in the treatment of aneurism, and which Hildebrandt himself first employed. Langenbeck's solution contained alcohol. Hildebrandt states that the solution used by him, as above, produced very little pain, and did *not* produce abscesses. The site selected by him for the insertion of the syringe was that part of the abdomen immediately over the tumor, although he remarked that the lower parts of the abdomen were more sensitive to puncture and injection than the parts around the umbilicus.

Guided by Hildebrandt's experience, I at first employed the solution recommended by him, and likewise selected the same portion of the abdominal wall for the place of puncture. However, I found that the injections *did* produce pain generally, and that occasionally it was very severe in character. Not unfrequently, too, they were followed by abscess. Under the impression that the presence of the glycerine might be the cause of these unpleasant effects, I next used a solution consisting of ergotine, 3 drams; distilled water, 9 drams. Of this solution, 12 minims were given with each injection. This, too, occasionally, was followed by pain and suppuration.

All of the solutions of ergotine that I had been using were turbid, and deposited a sediment on standing. Noticing this, I thought that possibly the inflammatory symptoms mentioned might be occasioned by the introduction into the cellular tissue of this insoluble portion of the ergotine. In a conversation upon the subject with Mr. J. P. Sharp, an accomplished druggist of this city, that gentleman assured me that all the ergotine found in the shops contained a small proportion of alcohol, and that it was impossible to procure a perfect solution of it without the aid of that vehicle. He suggested at the same time that an aqueous solution of ergot would probably answer my purpose, and be free from the objections I had found attending the use of ergotine. The suggestion has proved a most valuable one. He at once proceeded to prepare a solution according to the following form: "Fifty grains of the extract (Squibb's) are dissolved in 250 minims of water, the solution filtered and made up to 300 minims, by passing water through the filter to wash it, and the residue upon it. It represents ergot grain for minim, free from alcohol or other irritating substance."

Latterly I have used this solution exclusively, and thus far have seen no irritation, pain or inflammation result from it.

I no longer select the abdomen as the site for injection. Although some parts of the abdominal wall—as about the umbilicus, for example—may be less sensitive to puncture than others, yet all parts of it are more sensitive than the deltoid region; and inasmuch as this latter is more convenient, and the injections placed there equally efficacious, I now habitually select the arm in preference to any other part of the body.

Dr. J. H. Etheridge gave the details of treatment in one case, as follows:

*Hypodermic Use of Ergotine in Uterine Tumor—Probably Fibroid. A case illustrative of the good effects of such medication.*

Miss A. B. came under my care in June, 1873; æt. 16; has complained of great pain during menstruation for two and a half years; pain is paroxysmal, like labor; is of highly nervous temperament, well nourished; alimentary functions well performed; no trouble in defecating; no abdominal enlargement; kidneys functionate well, but micturition has been painful for many months. Physical examination: vagina small; introduction of finger difficult and somewhat painful; walls well lubricated; os normal; cervix rides high. Speculum repeals hyperæmic condition of cervix. Probe shows that there is retroflexion, a little toward the left side; depth normal. Upon reinserting finger, and passing it up between the uterus and bladder, a tumor, evidently pediculated and attached to the anterior wall of the uterus, and about the size of a large English hickory nut, was detected. It moved with the uterus. Diagnosis: subperitoneal fibroid tumor of the uterus. (Confirmed by Profs. Miller and Gunn, at subsequent examinations.)

Treatment: At first, stimulating applications were made to the cervix, as iodine, carbolic acid, silver, chromic acid, to excite absorption of the growth if possible. A fair trial of these, extending over eight weeks, produced no decrease in the tumor. Subcutaneous injections of ergotine were then resorted to, and fifteen or eighteen were given in all. At the end of that time (about forty days) the growth had disappeared about seventy-five per cent. The injections were then discontinued, because she was quite relieved from all symptoms on account of which she sought advice. She now menstruates painlessly, and is growing stronger in every particular.

Dr. H. P. Merriman reported the following cases from the clinic for diseases of women at the Mercy Hospital:

I. Mary K., a widow with three children, applied for treatment Oct. 2, 1873. She complained of pain in the back, head and side; dysuria; constipation; stomachic uneasiness and vomiting.

A small submucous fibroid tumor was discovered in the anterior wall of the uterus, by Dr. W. H. Byford, which had occasioned some leucorrhœa and metritis. Ergotine was used hypodermically from Oct. 8th to Jan. 7th, on alternate days, with the exception of the menstrual periods and one or two Sundays.

She was discharged "well" on the last date given, though some slight endometritis remained. Some remedies had been exhibited for the relief of the dysuria, and the urethra had been once dilated, after which this symptom in the case disappeared. Mineral acids had been employed as tonics and laxatives for the bowels. Three small abscesses had been produced by the ergotine, but were readily relieved.

She had experienced considerable pain during the early part of the treatment, but as improvement continued, the injections produced no discomfort. At first, small indurated wens were formed at the site of the injections, as large as filberts, but were discussed without difficulty.

The preparation used was rather a mixture than a solution. It was a saturated solution of the ergotine in alcohol, of which two minims represented one grain of ergotine, the amount used at each injection. Considerable sediment was deposited when the solution was undisturbed for a time.

The speaker regretted that he had lost sight of this patient, as she was a servant girl and had probably returned to service. He referred to the difficulty of keeping trace of out-patients, who usually ceased their attendance upon the Dispensaries as soon as benefit had been received.

II. Mary C., aged thirty-four years, married, without children, and having had no miscarriages, applied for treatment Feb. 5th. Has cephalalgia with lumbar and dorsal pain. Menses regular but profuse. Appetite fair, and normal stools. Says she has a "bunch in the side." On examination I found a subperitoneal fibroid tumor in the left side of the anterior wall of the womb, with a pedicle as large as a goose egg. The tumor could be distinctly recognized above the pubes, and was so far movable that its top could be depressed to the brim of the pelvis. Two grains of ergotine were injected beneath the skin every alternate day, except during menstruation, and after the fifth injection all pain disappeared, and the tumor ceased to enlarge. She thinks it is now somewhat smaller, and states that her health is better than it has been for two years. Up to the time of commencing the injections the tumor had been rapidly increasing. She experiences no pain from the injections beyond that of the prick of the syringe needle. In this case no abscess was formed, and small indurations which occurred, as in the previous case, disappeared in two weeks.

Vaginal examination now reveals the uterus in a normal position. There is slight swelling of the veins and the uterine mass seems immovable. Patient is still under treatment.

III. Honora McC., single, aged twenty-eight years, applied Feb. 28, complaining of pain in right side and dorsum; flashes of heat over forehead and eyes; restlessness and anorexia. Bowels are regular, menses normal as to time, but colorless and scanty. The hymen is present.

An extra-mural fibroid tumor was discovered on the left side of the womb, with a small pedicle. Injections of ergotine were at once commenced, and the tumor has gradually, but slowly, subsided. Slight dyspepsia was complained of, after about one week of treatment.

Dr. S. Fisher (the President) then relinquished the chair, and read the following details of a case occurring in his own practice:

*Case of Retroversion of the Uterus, Enlarged by a Submucous Fibroid Tumor—Uterus Replaced, and Tumor Discussed, by Hypodermic Injections of Ergotine.*

I first saw Mrs. S., Oct. 11, 1873. Aged 47 years; bilio-nervous temperament; has given birth to four children, the youngest eight years old; menstruation normal in regard to time, but rather profuse; complains of difficulty in urinating, and has found it necessary to have the bladder evacuated by the catheter a number of times; feels pressure on the rectum, preventing the free discharge of the feces. On making a vaginal examination I found the pelvis filled with a solid substance pressing upon the perineum, so that I could not pass my finger back into the vagina, without pressing the latter downwards. The os could only be found by passing two fingers high up behind the pubes; the os and neck seemed to be enlarged and flabby. As the patient was quite fleshy, pressure above the pubes did not assist in the diagnosis. However, I ventured to call it retroversion of the uterus, enlarged by a fibroid tumor. In order to satisfy both myself and the patient, of the exact nature of the case, before proceeding to operate, Prof. Byford was called the next day, to consult with me. He confirmed my diagnosis, and agreed with me in regard to the plan of reduction. The day after this, the 13th, I proceeded to replace the uterus by introducing a round piece of wood, about an inch in diameter, smooth and rounded at the end, into the rectum, making steady pressure against the fundus of the uterus, which soon began to yield to it, and in the course of twenty minutes it was pushed up three or four inches, putting the posterior part of the vagina on the stretch. The os at this stage could not be found, for by raising the fundus from the hollow of the sacrum to the promontory, it pressed the neck and os so high above the pubes that it could not be reached, showing that the uterus was much enlarged. I then introduced into the vagina a rubber bag, and inflated it with air, and left about two P. M. I was summoned to see her again at eight P. M. of the same day. She said that she had felt something give way internally about two hours previous to that time, producing great pain in the bowels, but was then easier. I made an examination and found the os in its normal situation, though quite open and flaccid.

On the 15th, I investigated the case thoroughly. The os was higher than usual and more open, so that I introduced my finger nearly an inch above the neck, and could feel the body of the uterus enlarged through the vagina. I passed the sound into the uterus three inches. I could now distinctly feel the uterine tumor above the pubes. and move it in any direction. It extended to a point half way between the umbilicus and pubes. The difficulty in voiding the urine and feces was entirely removed, and she was in every respect very comfortable. Debility and nervousness were only complained of. From this time, Oct. 15th, I saw her occasionally, and prescribed tonic remedies only, but did nothing specially for the uterine tumor until Feb. 4th. At that time the tumor appeared a little larger than when I examined it in October; it was movable, and she could feel it falling from side to side, as she moved. I then commenced the use of hypodermic injections of the following solution:

R. Ergotine, - - - dr. iij.
Glycerine, }
Water, } - - - *aa.* dr. vij.
M.

Injected hypodermically over the abdomen from thirty to forty drops of the above solution daily, according to effect, from the 4th to the 28th of Feb. The operation was performed every day at the same time (two P. M. to about four P. M.) She began at once to complain of pain in the back, with disagreeable sensations in the bowels, and occasional nausea. These symptoms generally continued more or less severe until the next morning. For the first few days after the injections were administered, she compared the sensation in the bowels to that of quickening in utero-gestation. After the first week, however, this motion ceased to be felt, but the uneasy feelings, pain and nausea continued as long as the injections were used, which was for twenty-four consecutive days. At the place where the point of the syringe was introduced the injection created tenderness, slight inflammation, and induration for the space of an inch or more in diameter in the surrounding tissues. She soon became quite nervous, and as it was time for menstruation to appear, the injections were omitted for one week. I then made an examination. The uterus appeared normal in size and position, but on account of the tenderness produced by the injections I could not examine satisfactorily above the pubes. For fear the tumor was not entirely discussed I continued the injections for five days, and then gave the same solution internally, in doses of forty drops daily, for a few days longer, which at first produced symptoms similar to those produced by the injections, but finally she felt no perceptible effect from them.

On the 19th of March, after the tenderness of the bowels had subsided, I made a thorough examination, by placing one finger on the os uteri, at the same time with the other hand making strong pressure above the pubes. By this method I could feel the size of the uterus, and it did not seem abnormal; the os and neck were also reduced in size to their normal condition. There was no abnormal discharge from the uterus, either before or during the treatment, which proves that the tumor must have disappeared by absorption, induced by the continued action of the uterus making constant pressure upon the tumor. I called to see her again April 9th, but she was absent on a visit to see her friends in Indiana. I ascertained, however, that she left in good health.

Drs. Etheridge, Jackson, and Walter Hay then discussed some questions connected with the subject presented.

Dr. Peck reported a case in which he had employed hypodermic injections of ergotine. The woman, an opium eater, had a flabby uterus, with symptoms of purulent metritis. An attack of angina pectoris supervened when the remedy was employed, but relief was immediate after its discontinuance.

Dr. Emmons gave the details of a case under his treatment in which hypodermic injections of ergotine were used in the discussion of a uterine tumor.

Considerable discussion ensued as to the *modus operandi* of the drug. Dr. Hollister believed that the results were due to its influence upon the vaso-motor nerves of the sympathetic system, and referred to recent observations made by him upon the iris in cases of cerebro-spinal meningitis, where ergotine had been administered.

Dr. E. Powell stated that in a case of double popliteal aneurism, in which the disease on one side had been relieved by digital compression, ergotine, although injected for a period lasting one month, had failed to change the condition of the parts. He added that the same remedy had been used for some time in the Woman's Hospital of New York, and that upon the occasion of his recent visit to that city, he had been informed that as much as 100 grains of the extract had been injected, without avail, for relief of salmucus fibroid tumors. This use of the drug had been consequently abandoned.

Dr. F. H. Davis then presented a specimen consisting of a kidney with greatly enlarged attached ureter, and heart enclosed in an ossified pericardium. He read from advance sheets of the *Medical Examiner* the ante and post-mortem history, and account of the microscopic examination of the degenerated tissues.

Dr. F. A. Emmons then presented the uterus of a woman who had died under the suspicion of having had an abortion produced upon her. A coroner's jury had rendered a verdict of death from uterine hæmorrhage. Dr. Emmons stated that Dr. R. H. Bingham believed an abortion had been produced. He himself believed the woman died from the effects of the uterine hæmorrhage. Dr. Simon expressed a dissenting opinion. He thought death had resulted from septicæmia. A purulent focus had been discovered in the substance of the cerebrum. Others existed in the uterine walls.

The lateness of the hour precluded further discussion.

Dr. Wilder requested suspension of the rules for the purpose of making a nomination for membership. Unanimous consent having been obtained, he nominated Mrs. Augusta P. Keat, a graduate of the "Woman's Hospital Medical College of Chicago."

The Secretary stated, that, while the Constitution did not expressly exclude the admission of women as members, certainly the precedent of such action had not yet been established, and he advised that the general principle be either adopted or rejected before the name of the candidate was referred to the Board of Censors.

Dr. Powell moved the passage of the following resolution:

"*Resolved*, That it is the sense of this Society that female physicians are ineligible for its membership.

Dr. Merriman moved to lay the resolution on the table, and his motion, after being seconded, was declared to be lost by the chair. A division of the house was called for, and the decision sustained.

The question recurring to Dr. Powell's motion, the resolution prevailed by a large majority.

The Secretary announced, that in addition to the paper of Dr. Montgomery, Dr. C. Paul Simon would present at the next meeting, in conjunction with Prof. DeLafontaine, a joint paper on "The Spectroscopic Properties of Old Blood," with demonstrations.

The Society then adjourned.

---

***Chicago Medical Society.*** Reported by WILL. T. MONTGOMERY.

Regular semi-monthly meeting, May 4, in the parlor of the Gault House. President Wm. E. Quine in the chair. Order for the meeting: Reports of cases and exhibition of pathological specimens.

The President reported a case of puerperal septicæmia that had occurred in the Cook County Hospital during the prevalence of puerperal fever, which seemed to afford striking evidence of the utility of local medication in the disorder.

The patient, a primapara, aged 20, was delivered, after a quick and easy labor, which resulted, however, in bad laceration of the perineum, of a healthy male child, weighing eight and a half pounds. The uterus did not contract very firmly, though an unusual amount of blood was not lost, and the after pains were not severe. On the second day after delivery, a copious secretion of milk was established, and the lochia was profuse, sanguinolent, and without offensive odor. In the evening castor oil was given by the nurse without orders from the physician. On the third day the patient seemed bright, vivacious and cheerful. She had had several movements of the bowels, and complained of nothing but intense pain in the forehead. Her eyes were red as though she had been weeping; the pupils contracted; tongue coated with a creamy fur; pulse 132, small and soft; respirations 36; temperature 104°. No pain in any part of abdomen, and no gaseous distention; uterus high and flabby; lochia dark brown, very profuse, and insupportably fetid; urine of normal character and quantity, though it had to be drawn off. During the day she experienced a succession of rigors, which were soon followed by increased acceleration of the pulse, elevation of temperature and profuse sweating. Quinine, gr. ij, and opium, gr. j, were directed to be given every three hours; sponge baths twice a day, and an intra-uterine bath of an aqueous solution of permanganate of potassium three times a day. The last-mentioned procedure was conducted in the following manner: The patient having been placed on a bed-pan, the disinfecting solution, strength 1 gr. to oz. i, to the amount of a pint, was allowed to flow into and out of the uterus through a double canula catheter from an ordinary nasal douche apparatus. No pain or inconvenience was complained of by the patient from the application. When the first application was made the pulse was 132. On the fourth day it was 128; on the fifth, 120; on the sixth, 118; on the seventh, 92; on the eighth, 82—the observations being taken at the same time of day. The pulse invariably fell from five to fifteen beats within two hours after the injections, though it rose quickly again, but only once above the original frequency. On the fifth day the patient seemed dull and listless; had no pain, and complained only of a feeling of soreness when pressure was made in right iliac region; diarrhœa and profuse sweating; her tongue was coated posteriorly with a creamy fur, while the tip and edges were very red, and the papillæ prominent. The abdominal walls were flaccid, and gurgling felt in right iliac fossa; uterus lower and firmer than before; the lochia abundant still, but much less offensive; urine normal in quantity. The previous prescription was now discontinued, and turpentine gtts. xv in emulsion was directed to be given every four hours alternately with quinine gr. ij, and hydrochloric acid gtt. iv. The patient being unable to retain either of the mixtures, they were omitted, and pills of quinine gr. ij, morphia gr. $\frac{1}{6}$, and strychnia gr. $\frac{1}{60}$, given every three hours. There was an abatement of all the symptoms corresponding with the decline in frequency of the pulse, and improvement in character of lochia, and on the eighth day she felt quite well and strongly desired food, though there was incomplete involution of the uterus.

The case presented two points of interest: it was one of pure uncomplicated septicæmia, and was unquestionably influenced more favorably by local than internal medication.

In the discussion of the case, Dr. Paoli, Vice-President of the Society, said he had found the sol. of permang. of pot. a very excellent one to use as an injection in offensive uterine discharges, but referred to the fact that uterine injections are condemned by most authors, on account of sometimes producing uterine colic. He thought it very difficult to distinguish between puerperal septicæmia and puerperal fever. Dr. Peterson thought the amount of force used in giving uterine injections had much to do in producing colic, and that the fluid should be passed in very gently. Dr. Stilliaus said he had experienced very satisfactory results from the use of uterine injections by means of the double canula catheter, and thought there was no danger of producing uterine colic when it was used. The President explained that uterine *injections* had not been given—only baths. He agreed with Dr. Paoli as to the difficulty of distinguishing between the various affections included under the head of puerperal fever. The latter term is used generically: in one instance applied to septicæmia, in another to phlebitis, in another to lymphangitis, in others to cellulitis, metritis, peritonitis, etc. It was not ordinarily difficult to tell when any one or more of these morbid states existed—generally several of them co-existed. The speaker then detailed the points of difference between the various disorders named, which would serve in establishing a diagnosis, and, in alluding to the etiology of puerperal fever, said that, while it occurred spontaneously and sporadically, there is a specific and infectious puerperal poison, which is not contagious, however, in the sense that variola poison is. The puerperal poison may give rise to one or more of several widely different morbid states, all of which, however, are included under the head of puerperal fever. One patient may contract a metro-peritonitis or septicæmia, or a cellulitis from another, who has neither of the disorders mentioned, but some other form of the disease. A patient may even contract a fatal puerperal fever from one laboring under any of the ordinary infectious diseases.

